# Optimum recording time of routine electroencephalogram for adults with epilepsy*

**DOI:** 10.3906/sag-1810-117

**Published:** 2019-04-18

**Authors:** Kemal TUTKAVUL, Yılmaz ÇETİNKAYA

**Affiliations:** 1 Clinic of Neurology, Haydarpaşa Numune Education and Research Hospital, University of Health Sciences, İstanbul Turkey

**Keywords:** Electroencephalography, epilepsy, time, probability, adult, humans

## Abstract

**Background/aim:**

This study aimed to reveal the optimum recording time of routine electroencephalogram (EEG) for adults with epilepsy.

**Materials and methods:**

In this clinical observational study we investigated features of paroxysms that emerged in EEGs recorded for 45 min in adults with epilepsy.

**Results:**

Paroxysms were detected in 38.14% of 97 patients. The probability of occurrence of paroxysm during the first 10 min was found to be statistically significantly low in comparison to the first 30 and 45 min (respectively P = 0.004, P = 0.0001). This probability was found to increase insignificantly when comparing the first 20 min with the first 30 min (P = 0.125), but it increased significantly in comparison to 45 min (P = 0.008). On the other hand, this probability was found to increase insignificantly when comparing the first 30 min with the first 45 min (P = 0.125). The cutoff point to specify the existence of interictal epileptiform discharges in the ROC analysis was found to be ≤39 min (95% CI: 0.958–1.000), and 90% of interictal epileptiform discharges were revealed during the first 30 min of EEG recording.

**Conclusion:**

The recording time of routine EEGs for adults with epilepsy should not be less 30 min.

## 1. Introduction

The recording time of electroencephalograms (EEGs) could be of critical importance in establishing the EEG findings associated with epilepsy. The American Clinical Neurophysiology Society guidelines suggest a minimum recording time of 20 min without artifacts to evaluate the wakefulness and recognize longer times for recordings with activation procedures and sleep [1]. The International League Against Epilepsy suggests a recording time of at least 30 min [2].

However, the recording times of routine EEGs vary from practitioner to practitioner. This could affect the expected yield of this test. Moreover, it cannot be estimated in which minute of a recording the EEG findings associated with epilepsy will emerge. Furthermore, features of patients or types of epilepsy may influence the detection of the mentioned EEG findings. 

Revealing the optimum recording time of routine EEG in a patient diagnosed with or suspected to have epilepsy can enhance the yield of this test. Therefore, this study aimed to reveal the optimum recording time of routine EEG for adults with epilepsy.

## 2. Materials and methods

### 2.1. Design of the study

In this clinical observational study we reviewed the demographical and clinical data of patients retrospectively and reevaluated EEG recordings that had already been done.

### 2.2. Sample

Data of patients were included in the study if they were diagnosed with epilepsy, were followed by the epilepsy outpatient clinic, were ≥18 years of age, came to their appointments consecutively, and gave informed consent. Breastfeeding mothers, patients who were pregnant, and those whose diagnoses were in doubt were excluded.

### 2.3. Analysis

Data from the files of the included patients were noted. The age, sex, age of first seizure, and duration of epilepsy were identified. The seizures and the epilepsy syndromes were classified as focal, generalized, and unidentified. Patients were grouped according to the seizure frequency (time between seizures) as frequent (≤1 month), occasionally (from 1 month to 2 years), and rare (>2 years). According to drug treatment, monotherapy, polytherapy, and ‘followed without drug treatment’ groups were determined. 

Routine EEGs were recorded during daytime office hours, on a full stomach, at wakefulness, with clean hair and for 45 min. The EEG recordings were done by silver cup electrodes connected through conductive paste and placed on the scalp according to the 10–20 system with Nicolet EEG Nicolet Ver. 5.11 equipment (Natus Neurology Incorporated, Middleton, WI, USA) and 0.5 Hz high-pass, 70 Hz low-pass, and 50 Hz notch filters were applied. As activation procedures eye opening and closing 10 times, 5 min of hyperventilation, and intermittent photic stimulation in the range of 5 to 30 Hz (eyes first opened then closed for 5 s respectively) were applied. These EEG recordings were reevaluated via bipolar and monopolar montages. 

### 2.4. Method of evaluation and statistics

In this study the term ‘paroxysm’ includes all interictal epileptiform and nonepileptiform graphoelement phenomena with sudden onset, rapid attainment of a maximum, and abrupt termination, distinguished from background activity [3]. The term ‘interictal epileptiform discharge’ (IED) incorporates generalized epileptiform discharges (GIED), and focal spikes and sharp waves (FIED).

The mean and standard deviations of patient age (years), age of first seizure (years), duration of epilepsy (years), time between the last fit and the recording (months), latency (minutes) to first paroxysm, IED, GIED, and FIED were calculated.

The correlations between the mean values of latency to first paroxysm, IED, GIED, and FIED and age, age of first seizure, and duration of epilepsy were examined with the Pearson correlation test. Sex and treatment by monotherapy or polytherapy were examined with the Mann–Whitney U test. Seizure frequency and epilepsy syndrome were examined with the Kruskal–Wallis test. 

The numbers of paroxysms detected during the first 10, 20, 30, and entire 45 min of recordings were registered. The correlation between increasing recording time intervals and the detected percentages of pathological activity was evaluated with McNemar’s test.

ROC analysis was done to determine the place in differential diagnosis from normal latency (minutes) to first IED.

NCSS (Number Cruncher Statistical System) 2007 Statistical Software (Logan, UT, USA) was used for statistical analysis. 

This study was approved by the local ethics committee of clinical studies (22 January 2018, number 2018 / 04). 

## 3. Results

Data of 97 patients were included in this study; 48.45% were male and 51.55% were female. Means of age, first seizure age, duration of epilepsy, and time between last fit and the recording were 33.66 ± 12.14 (18–66) years, 16.97 ± 10.9 (0.08–49) years, 16.9 ± 9.8 (2–44) years, and 21.54 ± 31.11(0–192) months, respectively. According to seizure frequency the frequent, occasional, and rare groups included 28 (28.87%), 32 (32.99%), and 37 (38.14%) patients, respectively. Seizures were classified as focal in 44 (45.36%), generalized in 50 (51.55%), and unidentified in 3 (3.09%) cases. Epilepsy syndromes were classified as focal in 48 (49.48%), generalized in 44 (45.36%), and unidentified in 5 (5.15%) cases. There were 54 patients on monotherapy, 42 on polytherapy, and 1 followed without drug treatment. Distribution of used drugs was 50.52% levetiracetam, 28.87% carbamazepine, 26.80% valproic acid, 26.80% pregabalin, 15.46% lamotrigine, 9.28% phenytoin, 7.22% topiramate, 6.16% zonisamide, 5.15% lacosamide, 3.09% oxcarbazepine, and 1.03% acetazolamide. 

**Figure 1:**
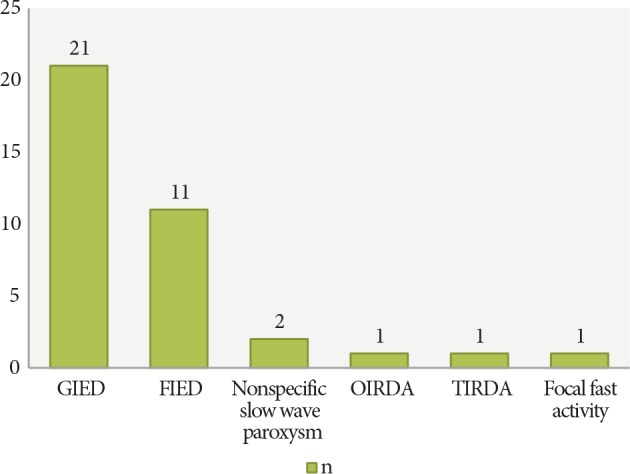
Spectrum of paroxysms (GIED = generalized epileptiform discharge; FIED = focal spikes and sharp waves; OIRDA = occipital intermittent delta activity; TIRDA = temporal intermittent delta activity).

The number of patients with paroxysms and IED on EEG were 37 (38.14%) and 32 (32.98%), respectively (Figure 1).

The mean values of latency (minutes) to first paroxysm, IED, GIED, and FIED were 32.92 ± 17.99, 33.83 ± 17.46, 37.25 ± 15.66, and 41.58 ± 10.62, respectively.

There was a correlation between age and age of first seizure and mean value of latency to first GIED (r = 0.201, P = 0.049; r = 0.260 P = 0.01, respectively), but not mean value of latency to FIED (P > 0.05, P > 0.05, respectively). A correlation was also not found between sex, epilepsy duration, seizure frequency, and mean values of latency to first IEDs (P > 0.05, P > 0.05, P = 0.283, respectively). 

Mean value of latency to first FIED was lower in the polytherapy group in comparison to the monotherapy group (P = 0.038), but mean value of latency to first GIED was not different between the polytherapy and monotherapy groups (P = 0.096).

It was observed that the probability of occurrence of paroxysms increased as the recording duration was longer. However, the correlation between them was not linear (Table 1).

**Table 1:** Recording time and the probability of catching paroxysms.

Recording time (min)	Paroxysms (%)	Correlation (P) between recording time (min) intervals (McNemar test)					
10	22.68	10–20	10–30	10–45	20–30	20–45	30–45
20	27.84	0.063					
30	31.96		0.004		0.125		
40	36.08			0.0001		0.008	0.125

Similarly, the probability of catching IEDs increased as the duration of EEG recording time was longer (Table 2). The existence of IED was evaluated with ROC analysis. To determine the place of latency (minutes) to first IED in differential diagnosis from normal, the area under the curve was found to be 0.998. The cutoff value was calculated with a sensitivity of 96.77%, a specificity of 98.48%, a positive predictive value of 96.8%, a negative predictive value of 98.5%, and a likelihood ratio (+) of 63.87 as ≤39 min (95% confidence interval: 0.958–1.000).

**Table 2:** Recording time and the probability of catching interictal epileptiform discharge (IED).

Recording time	IED (%)	Difference from10 min (%)	Difference from20 min (%)	Difference from30 min (%)
First 10 min	20.61			
First 20 min	25.77	5.16		
First 30 min	29.89	9.28	4.12	
Whole 45 min	32.98	12.37	7.21	3.09

**Figure 2:**
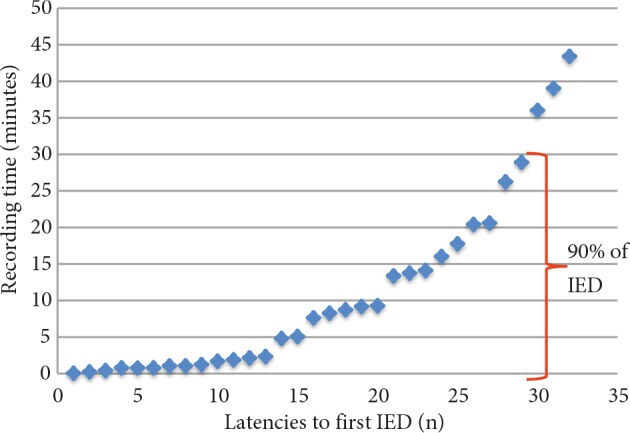
Distribution of latencies to first interictal epileptiform discharge (IED).

Finally, 90% of IEDs were detected during the first 30 min of the recordings and only 62% of IEDs during the first 10 min (Figure 2). 

## 4. Discussion

These results about catching paroxysms in routine EEGs in patients with epilepsy suggest that a recording time of 10 min is insufficient, 20 min is fair, 30 min is good, and more than 30 min is better. 

Burkholder et al. evaluated the probability of detecting IED in a cohort of adults and children in a 1-h recorded EEG and compared the first 30 min with the last 30 min [4]. They found that 44% of 1803 patients had a provisional diagnosis of epilepsy and 23.6% of all patients were found to have IED. This percentage was 43% in patients with a provisional diagnosis of epilepsy and 6.6% in patients with a nonspecific diagnosis. In 4.5% of all patients and 7.7% of patients with seizure-specific indications, IEDs were detected in the last 30 min. Similarly, our study (Table 2) shows that the probability to detect IED increases during a recording outlasting 30 min (3.09% more with 45 instead of 30 min). 

Theitler et al. studied the latency of IEDs under different situations [5]. They stated a cutoff value of 90% of all IEDs. The age range was 0.2–89 years. They investigated 316 routine EEGs (30 min) and 368 EEGs with sleep deprivation (60 min). Of these, 245 were from inpatients (96% after hospitalization because of seizures) and 439 from outpatients. The described cutoff was achieved in 14.6 min in routine EEGs, 21.3 min in EEGs with sleep deprivation, 14 min in patients after hospitalization because of seizure, and 21.3 min in ambulant patients. Our results (Figure 2) revealed that 90% of IEDs emerged in the first 30 min of routine EEGs. In the first 15 min only 71.8% of IEDs could be detected. The cause of this difference could be explained by the age range (≥18 years) of our patients. Also, in only 13 of 97 patients were the time intervals between the last fit and EEG recordings shorter than 15 days. 

Meritam et al. investigated the yield of EEGs [6]. Routine EEGs of 30 min and EEGs of 60 min during sleep were done for 303 patients aged 18–88 years and diagnosed with or suspected of having epilepsy. IEDs were detected in 31.68% of patients with routine EEGs. Our research (Table 2) likewise demonstrated that IEDs were detected in 29.89% of patients with routine EEGs in the first 30 min. 

This study’s results show that latency to first GIED is shorter than FIED, but seizure frequency, duration of epilepsy, and sex were not determinants of latency to first IED. These findings are consistent with the long-term EEG recording study results of Faulkner et al. and Werhahn et al. [7,8]. Also, our data reveal that age and age of onset of seizures could influence the latency to first GIED, and polytherapy the latency to first FIED. These findings are not consistent with others [7,8]. This difference may result from different age groups and the number and recording time of EEGs of the cohorts. 

The limitation of our study is that the recording time was confined to 45 min. Therefore, paroxysms that would emerge later could not be observed. Also, the number of patients should be higher to get more reliable data for subgroup analysis. 

In conclusion, the recording time of routine EEGs in adult patients diagnosed with epilepsy in outpatient clinics should not be less than 30 min. Short recordings may cause the loss of valuable data.
